# Multi-oil fat emulsion improves postoperative nutritional status and reduces complications in patients with hilar cholangiocarcinoma

**DOI:** 10.3389/fnut.2025.1628698

**Published:** 2025-09-16

**Authors:** Yang Tong, Sijia Bai, Ting Bi, Luyuan Jin, Yufu Tang, Yinghui Zhou, Xiaodong Feng, Wei Zhang

**Affiliations:** Department of Hepatobiliary Pancreatic Spleen Thyroid Surgery, General Hospital of Northern Theater Command, Shenyang, China

**Keywords:** hilar cholangiocarcinoma, multi-oil fat emulsion, nutritional status, inflammation complications, postoperative recovery

## Abstract

**Introduction:**

Hilar cholangiocarcinoma (HCCA) is a common and complex biliary tract tumor often accompanied by malnutrition and a high risk of postoperative complications. Effective nutritional support is critical for improving prognosis. This study aimed to investigate the effects of multi-oil fat emulsion on postoperative nutritional status, liver function, inflammatory response, and complication rates in patients with HCCA.

**Methods:**

A total of 88 patients with HCCA who underwent radical resection at our hospital between January 2022 and February 2024 were enrolled and randomly divided into two groups. The control group received 20% medium/long-chain fat milk injection, while the observation group received multi-oil fat milk injection, both combined with amino acid glucose solution for five consecutive days. Nutritional indices [albumin (ALB), hemoglobin (Hb), total protein (TP)], liver function indices [alanine aminotransferase (ALT), aspartate aminotransferase (AST), total bilirubin (TBIL)], inflammatory markers [C-reactive protein (CRP), tumor necrosis factor-α (TNF-α), interleukin-6 (IL-6)], and postoperative complications were assessed.

**Results:**

After intervention, both groups showed improved nutritional and liver function indices and reduced inflammatory markers compared with baseline (all P<0.01). The observation group demonstrated significantly higher ALB, Hb, and TP levels and lower ALT, AST, TBIL, CRP, TNF-α, and IL-6 levels than the control group (all *p* < 0.01). The incidence of complications was significantly lower in the observation group (4.54%) compared with the control group (20.46%) (*p* = 0.049).

**Discussion:**

Multi-oil fat emulsion provided superior benefits over conventional fat emulsion in enhancing postoperative nutritional status, improving liver function, and reducing systemic inflammation and complications in HCCA patients. These findings suggest that multi-oil fat emulsion may be a preferable option for postoperative parenteral nutrition in this population.

## Introduction

Hilar cholangiocarcinoma (HCCA) belongs to the mucosal epithelial carcinoma of the bile duct above the opening of the gallbladder duct, containing the common hepatic duct, the conjunctural part of the left and right hepatic ducts, as well as the left and right hepatic ducts (1). According to a large group of domestic case analysis reports, HCCA accounts for approximately 60 to 70% of biliary tract cancers and is one of the most common and complex biliary tract tumors (2). However, due to the influence of biological characteristics and anatomical location, the early diagnosis of HCCA is very low, and most patients are found at the advanced stage for the first time, missing the best treatment period, which increases the difficulty of treatment (3). Currently, surgical treatment is mainly used in clinical practice, and surgical methods are divided into radical and palliative. Usually, radical resection can achieve the best therapeutic effect (4). However, radical resection has a wide scope, and the treatment of the biliary tract is complicated to a certain extent. As a result, patients have a high risk of postoperative complications, resulting in increased risk of postoperative death, shortened survival period, and reduced quality of life in patients, which is not conducive to prognosis (5).

Cancer patients often suffer from malnutrition and are prone to postoperative inflammation, which in severe cases may even lead to death. Therefore, postoperative nutritional support based on parenteral nutrition is extremely important (6). Multi-oil fat emulsion is the fourth-generation fat emulsion. Its main components include soybean oil, coconut oil, olive oil, fish oil, and vitamin E. It is worth noting that the proportion of soybean oil in the formula has been reduced, while fish oil has been added, which conforms to the standard recommended by the World Health Organization of a ω-6/ω-3 ratio of 2.5:1 (7). Studies have shown that multi-oil fat emulsion can significantly alleviate the oxidative stress and excessive inflammatory conditions of patients after surgery, regulate immune function, thereby improving liver function and reducing infection complications (8).

In our study, we aimed to explore the influence of multi-oil fat emulsion on postoperative nutritional status and complications in HCCA patients.

## Data and methods

### General data

A total of 88 HCCA patients admitted to our hospital from January 2022 to February 2024 were selected as study subjects. Inclusion criteria included: (1) Patients met the diagnostic criteria for HCCA and underwent radical resection, (2) All patients were conscious and normal in comprehension, (3) Patients signed informed consent, (4) No abnormal fat metabolism; no fat milk allergy, and (5) No immunomodulators had been used recently. Exclusion criteria included: (1) Age < 18 years or > 85 years, (2) Patients had blood system diseases, (3) Patients had mental illness, (4) Patients had other vital organ diseases, and (5) Patients were unable to cooperate with researchers.

### Randomization and blinding

A group randomization design was adopted for random grouping. The random allocation sequence was generated by a computer. The allocation confidentiality measures were achieved through the use of sequential numbering, sealing, and opaque envelopes. After being deemed to meet the inclusion criteria, patients were randomly assigned to the control group or the observation group in a 1:1 ratio. This study was single-blind, and the participants were unaware of the allocation.

### Parenteral nutrition methods

Both groups of patients received parenteral nutrition support through a peripherally inserted central catheter after the operation. Patients in the observation group received an intravenous infusion of multi-oil fat milk emulsion (C6 ~ 24, Huarui Pharmaceutical Co., Ltd.) combined with amino acid glucose solution. Patients in the control group received an intravenous infusion of 20% medium/long-chain fat milk injection (C6 ~ 24, Huarui Pharmaceutical Co., Ltd.) combined with amino acid glucose solution. The two groups of patients received continuous intravenous infusion for 5 days. Both groups received parenteral nutrition support with an equal total caloric intake of 104.6 kJ/(kg·d) and an equal nitrogen intake of 0.2 g/(kg·d) after the operation. On the first day after the operation, the dosage of fat and glucose was halved. From the second to the fifth day after the operation, full nutritional support was provided, along with an adequate amount of electrolytes, vitamins, and trace elements, supplemented according to each kilogram of body weight.

### Observation indicators

The primary outcome measures of the study included nutritional status and liver function. For the measurement of nutritional status, 5 ml of venous blood was collected from patients upon waking in the morning and separated by centrifugation to obtain serum samples. Nutritional status indices, such as hemoglobin (Hb), serum total protein (TP), and serum albumin (ALB), were measured using an automatic biochemical analyzer. Besides, liver function indexes containing aspartate aminotransferase (AST), alanine aminotransferase (ALT), and total bilirubin (TBIL) were detected by an automatic biochemical analyzer.

The secondary outcome measures of the study included levels of inflammatory factors and the incidence of complications. Levels of inflammatory factors, including C-reactive protein (CRP) levels, were detected by turbidimetry, and serum tumor necrosis factor α (TNF-α) and interleukin-6 (IL-6) levels were detected by enzyme-linked immunosorbent assay (ELISA). Besides, the incidence of complications, including infection, biliary fistula, hemorrhage, and pleural effusion, was recorded in two groups.

### Statistical analysis

SPSS 19.0 software was implemented for statistical processing of the data. Statistical data were expressed as a rate (%), and Fisher’s exact test was used for comparison between groups. Measurement data were expressed as (x ± s), and the T-test was implemented for inter-group comparison. A *p*-value of < 0.05 was considered statistically significant.

## Results

### General data in two groups

**Figure 1:**
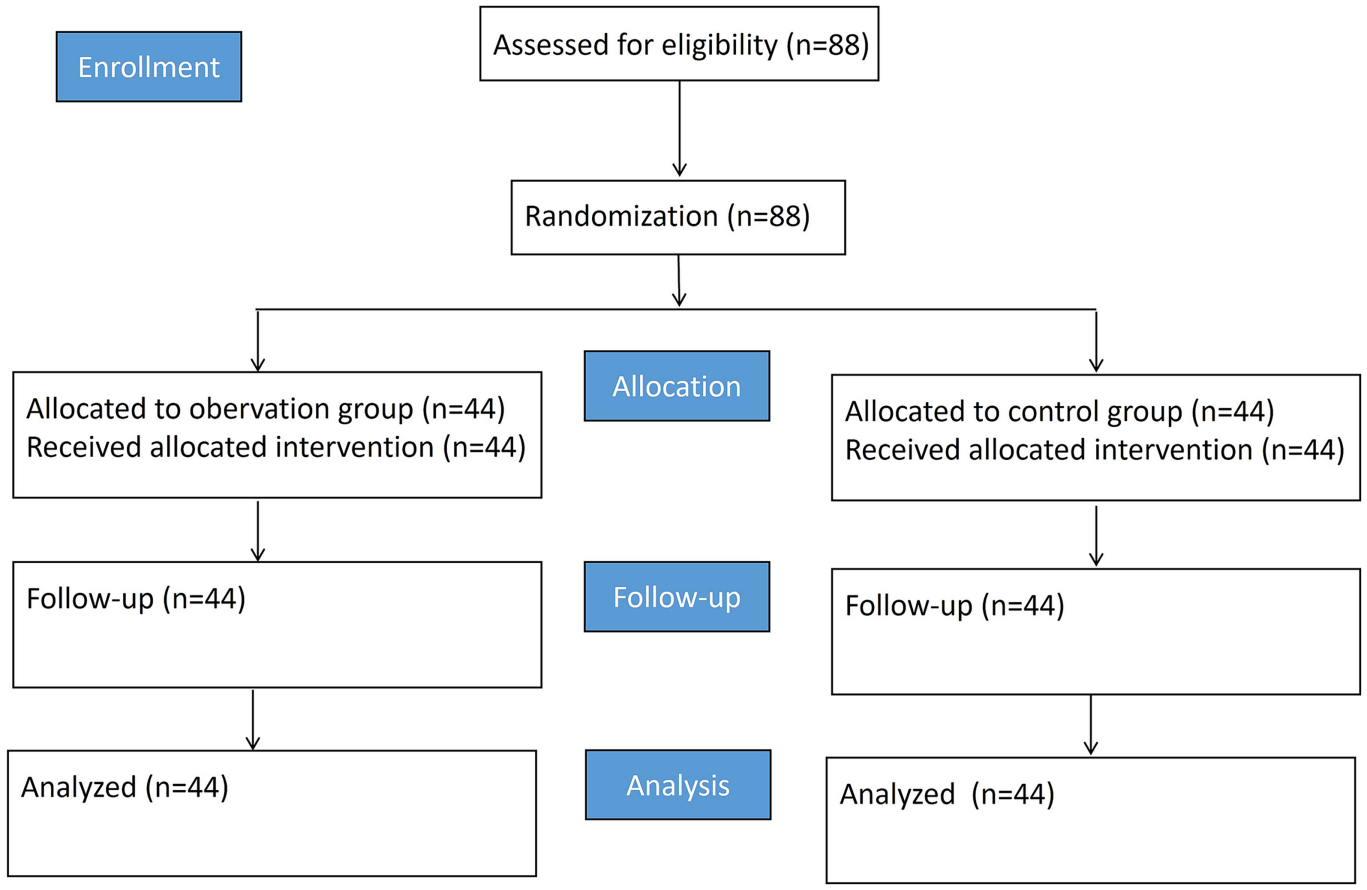
Flowchart of the study.

The flowchart of the study is shown in Figure 1. In the control group, there were 24 males and 20 females, with an average age of 49.26 ± 8.47 years. Among them, there were 23 cases of grade A and 21 cases of grade B. Meanwhile, there were 17 cases of stage I, 15 cases of stage II, and 12 cases of stage III. In the observation group, there were 25 males and 19 females, with an average age of 49.35 ± 8.51 years. Among them, there were 22 cases of grade A and 22 cases of grade B. Meanwhile, there were 18 cases of stage I, 16 cases of stage II, and 10 cases of stage III. There were no significant differences in general data such as gender, age, child grade, TNM stage, smoking history, drinking history, family history, and postoperative chemotherapy between the two groups (*p* > 0.05, Table 1), suggesting comparability.

**Table 1:** General data of patients in two groups.

Items	Control group (*n* = 44)	Observation group (*n* = 44)	Fisher	*p*
Gender				>0.999
Male	24 (54.55)	25 (56.82)		
Female	20 (45.45)	19 (43.18)		
Age (years)	49.26 ± 8.47	49.35 ± 8.51		0.960
Child grade				>0.999
Grade A	23 (52.27)	22 (50.00)		
Grade B	21 (47.73)	22 (50.00)		
TNM stage				0.926
Stage I	17 (38.64)	18 (40.91)		
Stage II	15 (34.09)	16 (36.36)		
Stage III	12 (27.27)	10 (22.73)		
Smoking history				0.670
Yes	23 (52.27)	20 (45.45)		
No	21 (47.73)	24 (54.55)		
Drinking history				0.830
Yes	25 (56.82)	23 (52.27)		
No	19 (43.18)	21 (47.73)		
Family history				0.828
Yes	19 (43.18)	17 (38.64)		
No	25 (56.82)	27 (61.36)		
Postoperative chemotherapy				0.822
Yes	30 (68.18)	28 (63.64)		
No	14 (31.82)	16 (36.36)		

### Nutritional status in two groups

**Figure 2:**
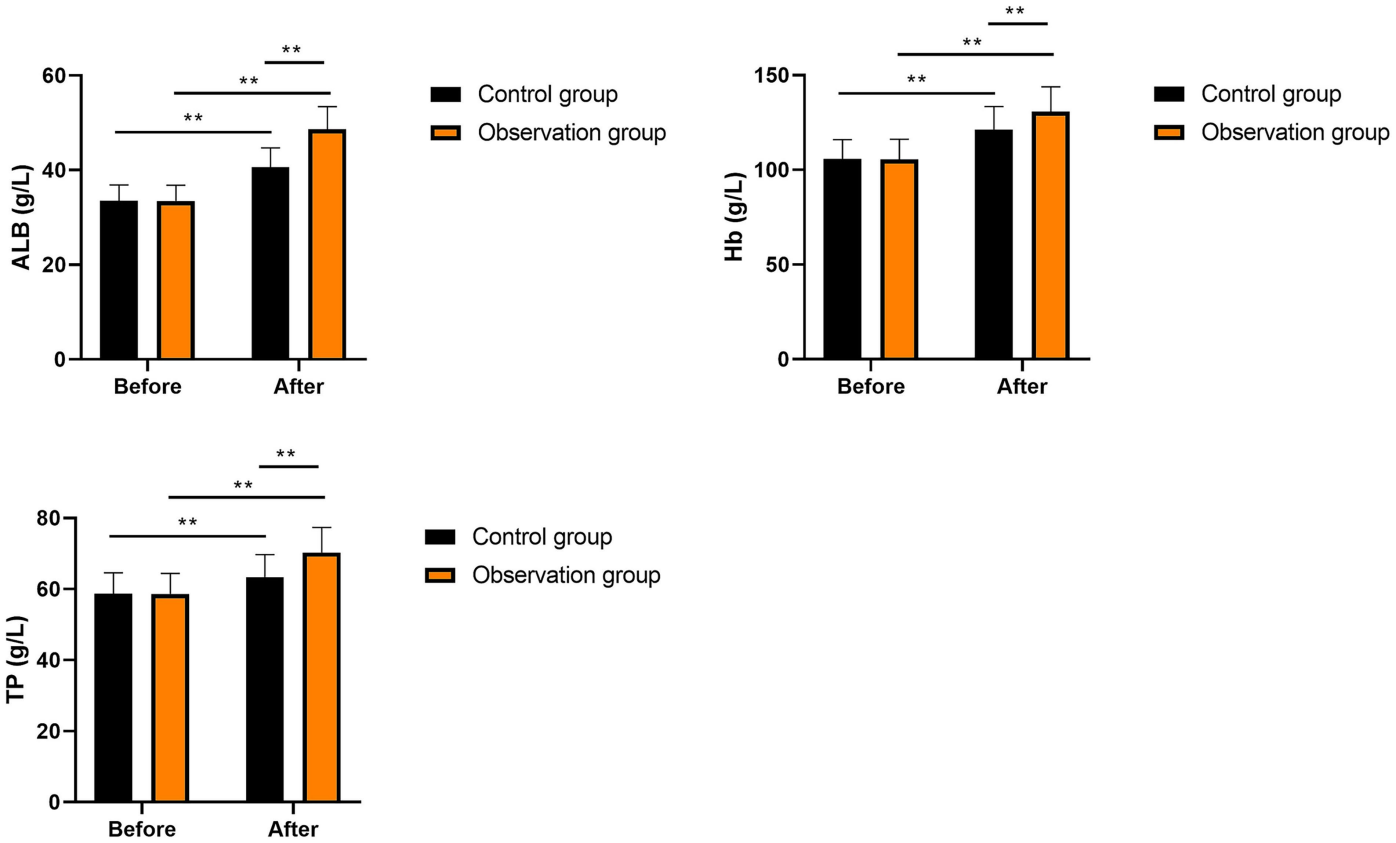
Nutritional status in two groups (^**^p < 0.01).

No significant differences were seen in ALB, Hb, and TP levels between the two groups prior to intervention (*p* > 0.05). After intervention, ALB, Hb, and TP levels were significantly elevated in the two groups (*p* < 0.01). Compared with the control group, the levels of ALB, Hb, and TP in the observation group were significantly higher after intervention (*p* < 0.01, Figure 2). These results suggest that multi-oil fat emulsion could improve the postoperative nutritional status of patients with HCCA.

### Liver function in the two groups

**Figure 3:**
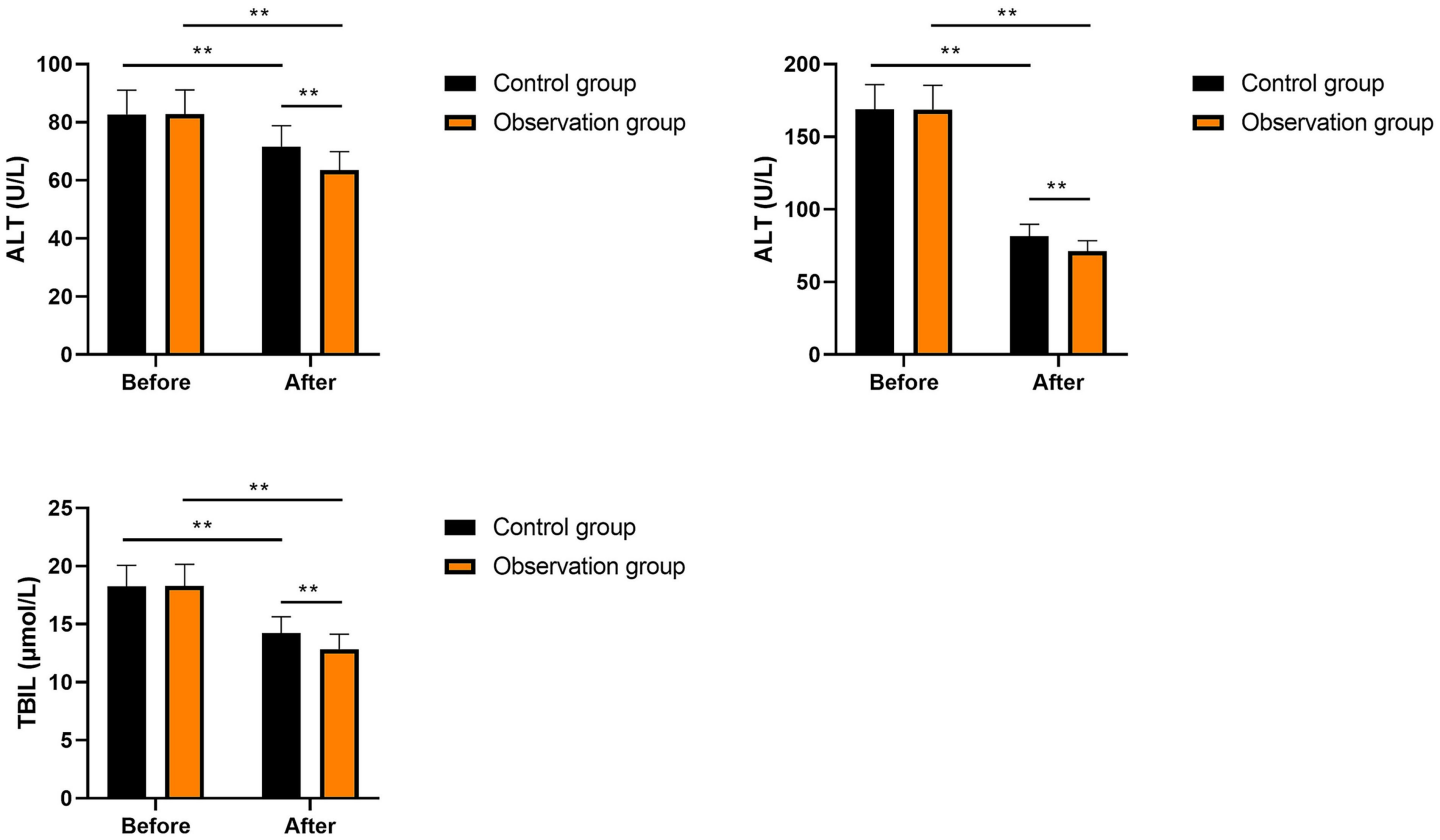
Liver function in two groups (^**^p < 0.01).

No significant differences were seen in ALT, AST, and TBIL levels between the two groups prior to intervention (*p* > 0.05). After intervention, ALT, AST, and TBIL levels were significantly diminished in the two groups (*p* < 0.01). Compared with the control group, the levels of ALT, AST, and TBIL in the observation group were significantly lower after intervention (*p* < 0.01, Figure 3). All these results suggested that multi-oil fat emulsion could improve the liver function of patients with HCCA.

### Inflammatory factors in the two groups

**Figure 4:**
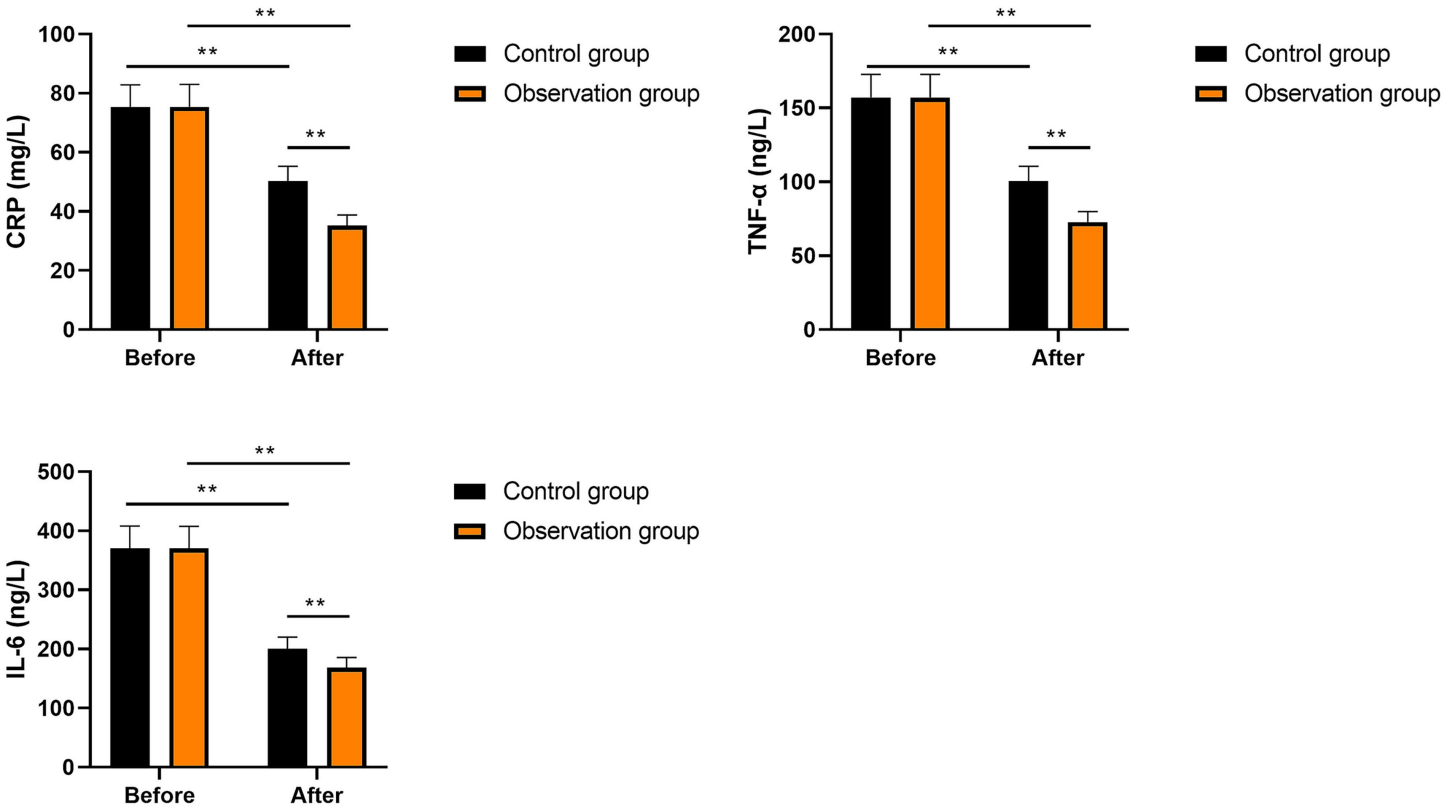
Inflammatory factors in two groups (^**^p < 0.01).

No significant differences were seen in CRP, IL-6, and TNF-α levels between the two groups prior to intervention (*p* > 0.05). After intervention, CRP, IL-6, and TNF-α levels were significantly diminished in the two groups (*p* < 0.01). Compared with the control group, the levels of CRP, IL-6, and TNF-α in the observation group were significantly lower after intervention (*p* < 0.01, Figure 4). These results suggest that multi-oil fat emulsion could alleviate the inflammatory response in patients with HCCA.

### Incidence of complications in the two groups

In the control group, there were three cases of infection, one case of biliary fistula, two cases of hemorrhage, and three cases of pleural effusion, resulting in a total incidence rate of 20.46% (9 of 44). In the observation group, there was one case of hemorrhage and one case of pleural effusion, resulting in a total incidence rate of 4.54% (2 of 44). Relative to the control group, the incidence of complications in the observation group was significantly lower (*p* < 0.05, Table 2). All these results suggested that multi-oil fat emulsion could reduce the incidence of complications in patients with HCCA.

**Table 2:** Incidence of complications in two groups.

Groups	Cases	Infection	Biliary fistula	Hemorrhage	Pleural effusion	Total incidence rate
Control group	44	3 (6.82)	1 (2.27)	2 (4.55)	3 (6.82)	9 (20.46)
Observation group	44	0 (0.00)	0 (0.00)	1 (2.27)	1 (2.27)	2 (4.54)
Fisher						
*p*						0.049

## Discussion

HCCA is one of the main types of extrahepatic cholangiocarcinoma, accounting for 65 to 75% (9). HCCA tends to invade the blood vessels, nerves, and lymphatic tissue of the portal area, resulting in a poor prognosis (10). It has been reported that HCCA, which cannot be surgically resected or radically resected due to its special anatomical location, has always been a significant concern for hepatobiliary surgeons (4). Recently, with the development of medical technology, the surgical treatment of HCCA has made some progress. After radical resection, most patients can effectively control the development of the disease and improve their survival rate by 3 to 5 years (11–13). HCCA is often associated with jaundice due to biliary obstruction, and 70% of patients with obstructive jaundice may have malnutrition after surgery (14). For patients after surgery, malnutrition is considered an important risk factor associated with postoperative complication rates and mortality (15). The radical resection will aggravate postoperative malnutrition due to significant trauma, long time, and postoperative stress, thus affecting the function of the body’s immune system, stimulating the production of TNF-α, IL-6, arachidonic acids, nitric oxide, and other inflammatory mediators in liver Kupffer cells, causing systemic inflammation and even biliary sepsis (16). Therefore, how to strengthen the postoperative nutrition of HCCA patients in a timely and effective manner, thereby repressing the inflammatory response of the body and reducing the postoperative incidence of complications, has become an important topic for hepatobiliary surgeons.

Early enteral nutrition is conducive to rapid postoperative recovery in tumor patients; during the empty period before enteral nutrition, parenteral nutrition can improve the prognosis of patients (17). As an important part of parenteral nutrition, fat emulsion is mainly used in ω-6 polyunsaturated fatty acids and soybean oil, and ω-6 polyunsaturated fatty acids can inhibit T cell-mediated immune function, increase postoperative inflammatory response, and inhibit immune function (18). Therefore, the selection of appropriate nutritional preparations plays a crucial role in promoting the overall recovery of patients after surgery.

Multi-oil fat emulsion is a balanced therapeutic fat emulsion containing four oils. These include 30% soybean oil (for essential fatty acids), 30% coconut oil (for quick energy), 25% olive oil (for monounsaturated fatty acids), 15% fish oil (for reducing inflammation and immune regulation), and 200 mg/L of vitamin E (for inhibiting lipid peroxidation) (19). Among them, fish oil is rich in ω-3 polyunsaturated fatty acids, mainly containing α-linolenic acid (ALA), docosahexaenoic acid (DHA), and eicosapentaenoic acid (EPA). ALA is an essential fatty acid. DHA has the function of stabilizing cell membranes, balancing cytokines and lipoproteins, and EPA is the precursor to prostaglandins and leukotrienes in the body (20).

In our study, the results revealed that after intervention, ALB, Hb, and TP levels were elevated in the two groups, with those in the observation group presenting higher levels compared to the control group, suggesting that multi-oil fat emulsion could enhance postoperative nutritional status in HCCA patients. The reason may be that multi-oil fat emulsion is a balanced therapeutic fat emulsion containing four kinds of oils. Fat milk rich in soybean oil can produce immunosuppressive effect on the body, increase the risk of infection, and affect the absorption of nutrients by the body, while the negative impact of multi-oil fat emulsion infusion on immune function is significantly lower than that of fat milk containing soybean oil, so patients can better absorb nutrients (21).

Our study also indicated that after intervention, ALT, AST, and TBIL levels declined in two groups, with those in the observation group presenting lower levels compared to the control group, suggesting that multi-oil fat emulsion could improve liver function in HCCA patients. It is because the fish oil in multi-oil fat emulsion is rich in ω-3 fatty acids, which can protect the liver. The high levels of ALA, DHA, and EPA can reduce linoleic acid metabolites, thereby reducing inflammatory mediators and hepatic damage (22). Meanwhile, multi-oil fat emulsion contains rich α-tocopherol, olive oil, vitamin E, and other antioxidants, which can reduce the oxidative stress of liver cells and protect liver function (23).

Besides, our study suggested that after intervention, CRP, IL-6, and TNF-α levels declined in the two groups, and those in the observation group presented lower levels when compared with the control group, suggesting that multi-oil fat emulsion could reduce the inflammatory response in HCCA patients. In general, ω-6 fatty acids in fat milk will produce inflammatory factors in the metabolism process, and too much will easily aggravate inflammation, while ω-3 fatty acids will inhibit inflammation in the metabolism process (24). Multi-oil fat emulsion can better reduce the body’s inflammatory response by adjusting the ratio of ω-6 to ω-3, reducing ω-6 and increasing ω-3 (25). Meanwhile, multi-oil fat emulsion is rich in EPA and DHA, which can not only compete and bind with arachidonic acid (AA), increase the contents of phospholipid EPA and DHA in immune cell membrane but also inhibit the activity of phospholipase PLA2 by decreasing the expression of cycpoxygenase-2 (COX-2), and reduce the arachidonic acid associated with AA, thus inhibiting inflammation and improving cellular immune function (26).

Our research has some limitations. First, our sample size is relatively small, which may lead to deviations between the data results and the actual values. Second, our research adopted a single-blind design, which inevitably resulted in subjective biases from the researchers, resulting in an imbalance in treatment between the two groups. Third, our research was a single-center study, and the sample was not representative, which may not accurately reflect the characteristics of a broader population. Fourthly, our research did not conduct long-term follow-up observations. The effects of multi-oil fat emulsion on the long-term postoperative nutritional status and complications in patients with HCCA are currently unclear. Therefore, more multi-center, double-blind, large-scale, and long-term studies should be conducted in the future to verify our findings further.

## Conclusion

Our study demonstrates that multi-oil fat emulsion can effectively improve postoperative nutritional status and liver function, while reducing inflammatory responses and the incidence of complications in HCCA patients.

## Data Availability

The datasets presented in this study can be found in online repositories. The names of the repository/repositories and accession number(s) can be found in the article/supplementary material.
